# How does family face relate to intention to seek therapist-guided and digital self-guided psychological interventions? mediating effects of interdependent stigma and help-seeking attitudes

**DOI:** 10.1007/s00127-025-02990-5

**Published:** 2025-09-22

**Authors:** Ben C. L. Yu, Floria H. N. Chio, Rebecca Y. M. Cheung, Kriti Kakani, Winnie W. S. Mak

**Affiliations:** 1 Department of Applied Social Sciences, The Hong Kong Polytechnic University, Hong Kong, China; 2 Department of Psychology, The Chinese University of Hong Kong, Hong Kong, China; 3 Department of Educational Studies, Xi’an Jiaotong-Liverpool University, Suzhou, China

**Keywords:** Interdependent stigma of help-seeking, Family face concern, Digital mental health, Cultures

## Abstract

**Purpose:**

The present study aimed to investigate the association between family face concern and help-seeking intention for therapist-guided and digital self-guided psychological interventions in four cultures, with possible mediation of interdependent stigma of help-seeking and attitudes towards seeking help.

**Methods:**

Using online questionnaires, six-hundred and forty-five responses (Mean age = 21.25, *SD* = 4.65; 70% women) were collected from college students in four regions, including Canada (*n* = 172), United Kingdom (*n* = 158), India (*n* = 160), and Hong Kong (*n* = 155). Levels of family face concern (adapted from the Face Concern Scale), interdependent stigma of help-seeking (Interdependent Stigma of Seeking Help Scale), attitudes towards therapist-guided and digital self-guided psychological intervention (adapted Face-to-Face Counselling Attitude Scale), intention to seek these interventions (items adapted to measure intention to seek help), and depressive symptoms (Patient Health Questionnaire-9) were assessed.

**Results:**

Using R (version 4.4.1) to conduct the path analysis, results showed that after controlling for depressive symptoms, family face concern was negatively associated with the intention to seek therapist-guided psychological intervention through the perception of higher social stigma on family members and negative attitudes towards the intervention. However, such a mediating effect was not significant for the intention to seek digital self-guided psychological intervention.

**Conclusions:**

The present study highlighted the potential negative influence of family face concern on one’s intention to seek psychological help. It also highlighted that digital self-guided psychological intervention may be less subject to the influence of family face concern and stigma.

Depression, a common mental disorder, has impacted an estimated 280 million individuals worldwide [1] and is associated with various adverse psychological consequences and tremendous disease burden [2–4]. Despite scientific advancement in the development of myriad forms of psychological treatments to treat depressive disorders and other common mental health conditions [5, 6], people around the world continue to express hesitancy in seeking professional mental health services [7, 8]. Even if interventions were sought, many individuals dropped out of treatment after a few attempts [9].

In recent decades, in addition to the conventional approach of delivering psychological interventions face-to-face by a therapist to a service user, various types of digital self-guided psychological interventions have burgeoned, demonstrating their efficacy and effectiveness in alleviating an array of common mental health conditions. In this study, therapist-guided psychological intervention refers to psychotherapy that is delivered in-vivo between the therapist and the service user. Digital self-guided interventions refer to the use of pre-programmed online psychological interventions on their own through mobile apps or websites, without the presence of any in-vivo therapist. Extant research supported the efficacy of both types of intervention in alleviating depressive symptoms [10, 11].

Among the numerous barriers identified [12, 13], cultural salient factors and stigma were found to be significant in deterring people’s help-seeking [14–17]. Notably, despite global efforts to tackle stigma, mental illness stigma, and help-seeking stigma remain pervasive worldwide [18, 19]. Individuals with mental health needs may not seek help due to concerns about how others may perceive them if they seek professional help [20, 21]. Among societies where face concern is prominent in the family [22, 23], individuals may be particularly mindful of how their help-seeking may reflect poorly on their family, thus discouraging them from seeking mental health services. While most studies focused on the individual’s own level of mental illness stigma and personal face concern on help-seeking intention [22, 23], few studies extended these concerns to the impact on individuals’ families. The present study aimed to examine how family face concern and interdependent stigma on help-seeking may be associated with individuals’ help-seeking attitudes and intention. The study also investigated the differences in relationships between these factors on help-seeking for therapist-guided interventions and digital self-guided interventions, given that the nature of these two types of treatments may pose different levels of threats to family face concern and interdependent stigma.

## Family face concern and help-seeking stigma

The concept of face plays a crucial role in understanding help-seeking stigma for mental health needs. Among different types of face concern, social face (mianzi), which relates to an individual’s status and respect gained through fulfilling social roles and personal accomplishments [24] may be particularly relevant. Seeking help for mental health conditions might be perceived as undesirable and socially unacceptable, given that there is pervasive social stigma associated with help-seeking [25]. This situation may be particularly pronounced in cultural contexts in which mental illness is considered a sign of weakness and lack of willpower [26] that may reflect poorly onto themselves and their families. Consequently, cultural contexts that value the preservation of family face concern may hold particularly negative views towards help-seeking and stigma towards families with members seeking professional help. This relationship between face concern and help-seeking stigma has been supported by studies indicating that face concern is positively associated with both public and self-stigma of help-seeking [27, 28].

However, the extant research has primarily examined the impact of personal face concern, focusing on individuals’ concern over their own face without considering the social face of their family. For individuals who espouse an interdependent self-construal, given that their sense of self is closely interconnected and defined by their relationships with in-group members, such as family [29, 30], family face concern may play a strong role in their consideration of seeking help for mental health conditions. Specifically, behaviors of people with higher levels of interdependent self-construal are more contingent on the thoughts, feelings, and actions of their significant others than their counterparts. In other words, significant others are accorded greater importance in the decision-making process of those with higher levels interdependent self-construal. Previous studies found that individuals living in Asian countries or in ethnic cultures that value families and kinships showed higher levels of interdependent self-construal than their Western or white counterparts [29, 31]. In the present study, family face concern is operationalized as the tendency to preserve the reputation and self-image of individuals’ family members and to avoid embarrassing their family members in front of others.

## Interdependent stigma of help-seeking and help-seeking tendency

In the past decades, researchers have put substantial efforts into studying how help-seeking stigma might impede help-seeking attitudes and behaviors [25, 32–34]. Given the pervasiveness of help-seeking stigma, people may avoid seeking help to prevent themselves from being evaluated negatively by others. They may prefer to conceal their help-seeking or keep this as a secret from their social circles. A recent meta-analysis synthesizing more than a hundred studies generally supported the negative association between help-seeking stigma and help-seeking attitude as well as help-seeking stigma and help-seeking intention [34]. Specifically, it was found that both public stigma and self-stigma of help-seeking were associated with poor attitudes towards help-seeking and lower intention to seek help [34]. However, the extant studies only examined the effects of the help-seeking stigma imposed on oneself, without extending this stigma onto their families.

Recently, to account for the effect of such help-seeking stigma associated with family members, [43] developed and validated a measure of interdependent stigma of help-seeking [43]. Interdependent stigma of help-seeking refers to an individual’s anticipated concerns about the repercussions that their seeking mental health services might have on their family members [43]. The construct of interdependent stigma highlighted the significance of considering the social images of family members in understanding help-seeking stigma, especially in cultural contexts where interdependent self-construal and family face concern are prevalent.

In their validation study, [43] found that interdependent stigma of help-seeking was associated with poor attitudes and reduced intention to seek help among college students from eight diverse countries and regions, including Hong Kong, Taiwan, Turkey, the United Arab Emirates, Germany, Australia, Brazil, and the United States [43]. These findings support the speculation that interdependent stigma may have a potential influence on individuals’ likelihood of seeking different forms of help for mental health conditions. Individuals with high levels of interdependent stigma may anticipate negative consequences for their family and internalize these concerns, shaping negative attitudes towards mental health services. It has been showed that when people perceived stigma in seeking help, they are more likely to have negative evaluations of help-seeking, resulting in less favorable attitudes [35]. Extensive research also demonstrates that higher levels of stigma are consistently associated with more negative attitudes towards seeking psychological help [21, 34–36], suggesting that interdependent stigma may also predict less favorable attitudes towards seeking help.

In addition, given that digital mental health interventions have burgeoned and demonstrated effectiveness in alleviating a number of common mental health conditions, including depression, help-seeking and stigma research should expand on their investigation beyond traditional in-vivo psychological interventions. Although previous studies found that individuals continue to prefer face-to-face psychological interventions over digital interventions [37, 38], these studies have not accounted for family face concern and interdependent stigma in their attitudes and intention. In cultural contexts where family reputation is highly valued and help-seeking stigma is pervasive, individuals may avoid in-vivo therapist-guided interventions to avoid being found out and prevent any potential negative impact their help-seeking may have on their family’s social status. In contrast, while digital self-guided interventions offer a level of anonymity and privacy that is not provided by therapist-guided interventions, seeking digital self-guided psychological interventions, which can be done at anytime anywhere, might be less susceptible to help-seeking stigma compared to therapist-guided interventions [39]. As such, we speculated that interdependent stigma of help-seeking might have a greater effect on therapist-guided interventions than on digital self-guided psychological interventions.

### The present study

**Fig. 1:**
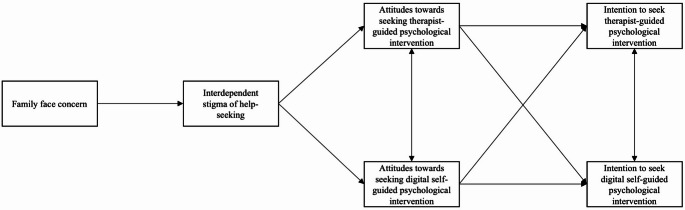
The proposed mediation model

The present study aims to examine the relationship between family face concern, interdependent stigma of help-seeking, attitudes towards help-seeking, and intentions to seek help for depression across four cultural contexts. We specifically investigated the potential mediating effects of interdependent stigma of help-seeking and help-seeking attitudes on the association between family face concern and help-seeking intention (Fig. 1). Our research covered both therapist-guided and digital self-guided psychological interventions, aiming to provide a comprehensive understanding of how interdependent stigma might influence help-seeking behaviors across different modalities. It was hypothesized that family face concern would be associated with lower intention to seek psychological intervention through higher interdependent stigma and poor attitudes towards psychological intervention. It was also hypothesized that the indirect effect of family face concern on intention to seek digital self-guided psychological intervention through interdependent stigma and attitudes towards digital self-guided psychological intervention would be smaller than the therapist-guided ones.

## Method

### Participants

By assuming a moderate effect size on the indirect effects from family face concern to the outcomes through interdependent stigma and attitudes towards seeking therapist-guided or digital self-guided interventions, at least 160 participants would be needed to achieve a power of 80% with an alpha level of 0.05 [40]. Nine-hundred and sixty-five participants were first recruited from universities of four cultural contexts, including, Hong Kong, India, Canada, and United Kingdom. These four countries/regions were selected because they represent varying levels of collectivism based on House et al., (2004)’s cross-cultural study on societal in-group collectivism practice score, which measures the extent to which a culture expresses loyalty and interdependence within families [41]. House et al. (2004) computed a continuous score for each of the 61 cultures studied, which were then categorized into three bands representing significantly different levels of scores. India was categorized in Band A (highest in collectivism), Hong Kong in Band B (middle level), and Canada and England in Band C (lowest in collectivism). These four cultural contexts ensure the coverage of cultures with varying degrees of collectivism-individualism and emphasis on family interdependence, which is highly relevant in the present study examining family face concern and interdependent stigma. Among them, 645 participants (70% women, mean age = 21.25 years) provided valid responses. The validation check consists of five attention check items designed to assess participant attentiveness and ensure data quality. A sample item is “please indicate strongly disagree for this question”. Participants who answered all five items correctly were considered attentive. Only data from those who passed all five checks were retained for the main analyses. This procedure was implemented to minimize the inclusion of inattentive or careless responses, which could compromise data validity. Table 1. shows the demographic information of the participants across cultures in detail.

### Procedure

In Hong Kong, participants were recruited through a subject pool and mass mail system in a public university located in Sha Tin. In Canada and United Kingdom, participants were recruited through the subject pool in a public university of each cultural context in Peterborough and Reading, respectively. In India, participants were recruited in a public university through snowball sampling. The participants in India were mainly from four regions, including Raipur, Delhi, Kochi, and Bangalore. After providing informed consent, participants were asked to complete an online questionnaire in Qualtrics, an online survey platform. For participants recruited through subject pool, their efforts were compensated by giving a course credit; for participants who were not recruited through subject pool, they were compensated with ~ USD 6.3 and ~ USD 4.7 in Hong Kong and India, respectively, to reflect differential cost of living in the respective regions. Ethics approval was obtained from the ethics committees of universities in Hong Kong, Canada, and the UK. Additional ethics approval from the Indian university was not sought, as the approval already granted by the Hong Kong institution was considered sufficient to meet the ethical standards required by the university in India.

### Measures

#### Interdependent stigma of help-seeking

 The Interdependent Stigma of Seeking Help Scale [43] was used to measure individuals’ anticipated concerns about the impact of their seeking mental health services for depression on family members. Responses were recorded on a 5-point Likert scale ranging from 1 (not at all) to 5 (a great deal), with higher composite scores reflecting greater perceptions of the interdependent stigma against seeking help. The scale was validated in eight countries/regions and demonstrated satisfactory psychometric properties [43]. In the present study, the scale showed satisfactory internal consistency (Cronbach’s alpha = 0.93).

#### Attitudes towards therapist-guided psychological intervention

 The 10-item Face-to-Face Counselling Attitude Scale [44] was adapted to measure attitudes towards therapist-guided psychological intervention and self-guided digital psychological intervention, using a 6-point Likert scale ranging from 1 (strongly disagree) to 6 (strongly agree). Before participants responded to the items, we provided definitions for therapist-guided and digital self-guided psychological interventions. Therapist-guided intervention was defined as face-to-face mental health services accessed either in person at a counselor’s office or through teleconferencing. Digital self-guided intervention was described as the use of pre-programmed online psychological interventions via mobile apps or websites, without the real-life presence or teleconferencing support of a therapist. Participants completed the items for both types of counseling, with each type of intervention comprising 10 items. Sample items for therapist-guided and digital self-guided psychological interventions include, respectively, “If I were having a personal problem, seeking help with face-to-face psychotherapy would be the last option I would consider” and “If I were having a personal problem, seeking help with a digital self-guided intervention would be the last option I would consider.” The scales’ composite scores were used in the present study, with higher scores indicating a more positive attitude. The internal consistency of both scales is satisfactory in the present study (Cronbach’s alphas = 0.87 for both scales).

#### Intention to seek therapist-guided and digital self-guided psychological intervention

Three items, adapted from Mo and Mak [45] were used to measure one’s intention to seek therapist-guided and digital self-guided interventions on a 6-point Likert scale ranging from 1 (Strongly Disagree) to 6 (Strongly Agree). The sample items included “If I have depression, I intend to seek face-to-face psychotherapy” (for therapist-guided intervention) and “If I have depression, I intend to seek digital self-guided intervention.” Each type of intervention is represented by three items, with higher scores on the scale indicating greater levels of intention to seek help. The internal consistency of the scales for both therapist-guided and digital self-guided interventions was satisfactory. The Cronbach’s alphas for intention to seek therapist-guided and digital self-guided interventions were 0.91 and 0.95 respectively,

#### Depressive symptoms

The 9-item Patient Health Questionnaire [46] was used to measure the intensity of depressive symptoms on a 4-point Likert scale ranging from 0 (not at all) to 3 (nearly every day). It has been validated across cultural contexts with good reliability [47, 48]. A sample item from the PHQ-9 is “Feeling down, depressed, or hopeless.” The internal consistency of the scale is satisfactory in the present study (Cronbach’s alpha = 0.87).

#### Sociodemographics and mental health history

Participants were asked to self-report their gender (man, woman, transgender, prefer not to say), age (in years), and ethnicity. Participants were also asked to indicate whether they had ever been diagnosed with a mental illness (Yes/No).

### Data analysis

Since the family concern scale has not been validated previously, exploratory factor analysis (EFA) and confirmatory factor analysis (CFA) were performed using IBM SPSS Statistics version 29 and R version 4.4.1 on the scale, respectively, prior to the mediation analysis. Before the factor analyses, the data was randomly split into two halves for EFA (*n* = 322) and CFA (*n* = 323). For EFA, suitability of the data for structure detection was examined by Kaiser-Meyer-Olkin measure of sampling adequacy (KMO) and Bartlett’s test of sphericity, with a KMO value of at least 0.6 for good factor analysis and a statistically significant Barlett’s test [49]. After checking the suitability of the data for factor analysis, principal axis factoring with direct oblimin rotation was conducted. Direct oblimin rotation was used to allow for correlations among factors, which is recommended to result in a more accurate solution [49, 50]. Principal axis factoring (PAF) was chosen as the extraction method because it estimates underlying latent structures by focusing on the common variance shared among observed variables. This approach is recommended when the research objective is to identify latent constructs [51]. Items were selected when they had factor loading larger than 0.4 on the primary factor, had factor loading below 0.30 on the alternative factor (if any), and had a difference greater than 0.20 between the primary and the alternative factor loadings [52].

CFA was then performed on the factor structure suggested by the EFA using the lavaan package [53]. The model fit was assessed based on the goodness-of-fit indices, including the comparative fit index (CFI), Tucker-Lewis index (TLI), root mean square error of approximation (RMSEA), and standardized root mean squared residual (SRMR). The following fit criteria were used: CFI ≥ 0.95, TLI ≥ 0.95, R MSEA ≤ 0.06 and SRMR ≤ 0.08 for good fit, and CFI ≥ 0.90, TLI ≥ 0.90, RMSEA ≤ 0.10 and SRMR ≤ 0.10 for acceptable or marginal fit [54, 55].

Measurement invariance of the Face Concern Scale was tested across the four cultures using multigroup CFA. Specifically, configural invariance (equivalent factor structure), metric invariance (equivalent factor loadings), and scalar invariance (equivalent factor loadings and intercepts) were examined. At each step, model fit was compared to the less constrained model using changes in CFI and RMSEA. Following established guidelines, invariance was considered supported if the decrease in CFI (ΔCFI) was ≤ 0.01 and the increase in RMSEA (ΔRMSEA) was ≤ 0.015 [56, 57].

Finally, path analyses were performed in R to investigate the mediating role of the interdependent stigma of help-seeking and attitude towards (therapist-guided and digital self-guided) psychological interventions in the association between family face concern and intention to seek (therapist-guided and digital self-guided) psychological intervention. The indirect effects were estimated using the manymome package with 5,000 bootstrapped samples [58]. Analyses were first conducted using the aggregate sample to provide an overall estimate of mediation effects. Given the theoretical importance of cultural orientation, we also grouped Hong Kong and India as collectivistic cultures and the UK and Canada as individualistic cultures. The groupings are supported by cross-cultural research showing that Hong Kong and India score more towards collectivism, whereas UK and Canada score more towards individualism [41, 59]. Grouping countries by cultural orientation, rather than analyzing all four groups separately, increases statistical power and allows for a more focused test of whether the mediation pathways differ by cultural orientations.

Path analyses were conducted using both the aggregate sample and separately within each of the two cultural groups (i.e., collectivistic and individualistic group). The aggregate analysis provided an overall estimate of mediation effects, while the culture-specific analyses allowed for the examination of mediation pathways within each group. To assess whether the strengths of the indirect effects differed across cultures, Wald tests were used to compare the parameter estimates. In the path analyses, family face concern was structured as the independent variable; interdependent stigma of help-seeking and attitudes towards therapist-guided and digital self-guided psychological intervention were treated as first and second mediators, respectively, while the intention to seek therapist-guided and digital self-guided psychological intervention were structured as dependent variables. All possible direct effects and covariances were also structured, which resulted in a saturated model (perfect goodness-of-fit). Significant indirect effect constitutes significant mediation effect. Figure 1 shows the tested model. Since interdependent stigma and intention to seek interventions are specific to depression, the composite score of PHQ-9 was controlled in the model to account for the potential effect of depressive symptoms. 

#### Family face concern

Twelve items were drafted to measure family face concern based on the Face Concern Scale [42], given that, to the authors’ knowledge, there is no scale tapping into face concern of family members available in the literature. The original Face Concern Scale [42] was a 34-item scale developed to measure how individuals negotiate face during interpersonal conflict. In the present study, we first attempted to modify the items of the face concern scale to specifically address family members’ face concerns. We then removed items that were repetitive or did not fit the family context. For example, the item “I was concerned with not bringing shame to the other person” was modified to “I was concerned with not bringing shame to my family members.” Ultimately, 12 items were created to measure family face concern on a 5-point Likert scale from 1 (Strongly disagree) to 5 (Strongly agree). Table 2 displays the items of the Family Face Concern Scale. Given that the results of factor analyses (see Data Analysis and Results sections) suggested a unidimensional structure, a composite score of the scale was used, with higher scores indicating greater concern over the social face of family members. The internal consistency of the scale was found to be satisfactory (Cronbach’s alpha = 0.92).

## Results

### Descriptive analyses

Table 3 shows the mean levels of each variable across cultures. Normality of the data was assessed using skewness and kurtosis. All variables met the criteria for normal distribution, falling within the recommended cutoffs of ± 2 for skewness and ± 7 for kurtosis [60]. ANOVAs were conducted to compare the mean differences in all the variables of interest across countries/regions. We observed significant group effects for all the variables of interest, except for the intention to seek digital self-guided psychological intervention among cultures. Specifically, as reflected by the post-hoc tests, for family face concern, the mean level in India was significantly higher than in the other three cultures. For interdependent stigma of help-seeking, people in Hong Kong and India showed higher levels than those in Canada and the UK, while no significant difference was observed between Hong Kong and India or between Canada and the UK. The observed differences in these two cultural variables among the four cultures are consistent with the societal in-group collectivism practice scores reported by House et al. (2004) [41]. Participants from India were expected to score the highest, followed by those from Hong Kong, and subsequently by those from the two Western countries.

**Table 1:** Demographic information of participants across the four cultures

Demographic	Hong Kong	Canada	United Kingdom	India	Total
**Age**	20.40 (2.79)	21.36 (6.15)	20.71 (3.82)	22.47 (4.71)	21.25 (4.65)
**Gender**					
Woman	108	139	134	71	452
Man	46	26	18	85	175
Transgender	0	3	1	0	4
Prefer not to say	0	2	2	3	7
**Ethnicity**					
African	0	13	6	0	19
Canadian	0	93	1	0	94
Chinese	63	3	6	0	72
English	0	4	80	1	85
First Nations or Indigenous or Aboriginal	0	3	0	0	3
Hongkonger	75	0	1	0	76
Hispanic	0	1	0	0	1
Indian	1	16	14	155	186
Irish	0	1	1	0	2
Latinx	0	0	1	0	1
Scottish	0	1	0	0	1
Welsh	0	0	6	0	6
Other	7	16	22	0	45
Mixed	4	17	15	1	37
Prefer not to answer	4	2	1	2	9
Don’t know	0	1	3	1	5
**Mental illness history**					
Yes	29	63	46	21	159
No	125	108	111	139	483

Mean (standard deviation)

**Table 2:** Factor loadings of Family Face Concern Scale

	EFA	CFA
1. I was concerned with not bringing shame to my family members	0.60	0.48
2. Relationship harmony with my family members was important to me	0.56	0.54
3. Helping to maintain the family members’ pride was important to me	0.77	0.79
4. I wanted to maintain a credible image of my family members in front of other person	0.79	0.80
5. I didn’t want to embarrass my family members in front of the other person	0.78	0.74
6. I tried to be sensitive to my family members’ self-worth	0.58	0.64
7. I wanted to maintain the dignity of my family members in front of other person	0.80	0.82
8. I wanted the other person to show my family members proper respect	0.63	0.61
9. Preserving the self-images of my family members was important to me	0.79	0.85
10. Saving my family members’ face was important to me	0.84	0.85
11. I was concerned with not making my family members to appear weak in front of the other person	0.72	0.76
12. I don’t want my family members to look incompetent	0.71	0.75

EFA = factor loading of exploratory factor analysis. CFA = factor loading of confirmatory factor analysis

For attitudes towards therapist-guided psychological intervention, people in Hong Kong had significantly poorer attitudes than those in Canada and the UK, while people in India had significantly poorer attitudes than those in the UK. For attitudes towards digital self-guided psychological intervention, the only significant difference observed was between India and Canada, with people in India showing poorer attitudes. For the intention to seek therapist-guided psychological intervention, the only significant difference was found between Hong Kong and the UK, with people in the UK showing greater intention.

Paired-sample t-tests were also conducted to examine the mean differences between attitudes towards therapist-guided and digital self-guided psychological interventions and between intention to seek these two types of interventions. Significant results were observed that the mean levels of both attitudes [Mean difference = 0.32, SD = 1.15, t(644) = 7.02, *p* <.001] and intention to seek therapist-guided psychological intervention [Mean difference = 0.87, SD = 1.64, t(643) = 13.41, *p* <.001] higher than digital self-guided ones. This implies that, in our samples, therapist-guided psychological intervention is preferred over digital self-guided ones.

### Factor analyses for family face concern scale

As to EFA for the Family Face Concern Scale, the results indicated that the KMO was 0.93, indicating a high proportion of variance among the variables that could be due to underlying factors. The Bartlett’s test of sphericity was also significant (*p* <.001), suggesting a rejection of the null hypothesis, which posited that there are no related or suitable variables in the correlation matrix for structure detection. Both indices converged that the data were suitable for EFA.

The results of the EFA suggested a unidimensional factor structure, with all items loaded on a single factor. A factor loading of 0.40 was adopted as the minimum criterion for item retention [52] and all factor loadings ranged from 0.56 to 0.84. The one-factor model could explain 55.623% of the total variance of the items. Therefore, a unidimensional factor structure was constructed in the CFA for the Family Face Concern Scale with all 12 items loaded on one single latent factor.

CFA was conducted on the second half of the sample to evaluate the factor structure of the Face Concern Scale identified from the exploratory factor analysis. The initial model showed an acceptable fit to the data: χ²(54) = 213.86, *p* <.001; CFI = 0.93; TLI = 0.92; RMSEA = 0.096; SRMR = 0.04. Modification indices suggested that adding covariances between some pairs of items. As suggested, covariance between items can indicate conceptual similarity [61]. After scrutinizing the items, error covariances were added between two pairs of items (item 5 and 7, item 4 and 5) that had similar wordings and conceptual similarity. After including these correlated error terms, the revised model demonstrated a better fit: χ²(52) = 159.79, *p* <.001; CFI = 0.96; TLI = 0.94; RMSEA = 0.08; SRMR = 0.04. All factor loadings in the final model were statistically significant (*p* <.001) and ranged from 0.48 to 0.85, supporting the construct validity of the scale.

To assess whether the Face Concern Scale operated equivalently across the four cultural groups, multi-group CFA was performed on the entire sample (*N* = 645) using the final model. Configural, metric, and scalar invariance were tested sequentially. The configural invariance model, which allowed all parameters to be freely estimated across groups, demonstrated an acceptable marginal fit: χ²(208) = 537.83, CFI = 0.93, TLI = 0.91, RMSEA = 0.099, SRMR = 0.05. While RMSEA falls within the marginal fit (48–49), considering the fit indices of CFI, TLI, and also SRMR, the model is considered to be acceptable. When factor loadings were constrained to be equal across groups (metric invariance), model fit remained acceptable, with ΔCFI = 0.008 and ΔRMSEA = 0.002 compared to the configural model. Further constraining item intercepts (scalar invariance) resulted in ΔCFI = 0.011 and ΔRMSEA = 0.001 relative to the metric model. The change in CFI slightly exceeded the recommended cutoff by 0.001, while the change in RMSEA remained well within the accepted threshold. Considering the overall pattern of fit indices, these results provide reasonable support for scalar invariance across the four groups.

### Mediation analysis

Table 4 shows the zero-order correlations among the variables of interest. It was found that family face concern was significantly associated only with higher interdependent stigma of help-seeking (*r* =.21, *p* <.001) and higher intention to seek digital self-guided psychological intervention (*r* =.09, *p* =.018). However, the strength of the correlation between family face concern and intention to seek digital self-guided psychological intervention was very small. Moreover, the interdependent stigma of help-seeking was significantly associated only with poor attitudes towards therapist-guided psychological intervention (*r* = −.24, *p* <.001). Attitudes towards therapist-guided psychological intervention (*r* =.66, *p* <.001) and digital self-guided psychological intervention (*r* =.61, *p* <.001) were strongly correlated with the intention to seek the corresponding type of intervention.

**Table 3:** Means and standard deviation (in parenthesis) across the four cultures

Demographic	Hong Kong	Canada	United Kingdom	India	Total	F(df)	*p*-value	Skewness	Kurtosis
Family face concern	3.71 (0.59)	3.70 (0.79)	3.57 (0.81)	3.93 (0.70)	3.73 (0.74)	6.83 (3, 641)	< 0.001	− 0.85 (0.10)	1.11 (0.19)
Interdependent stigma of help-seeking	2.16 (0.84)	1.71 (1.44)	1.73 (0.86)	2.08 (0.86)	1.91 (0.87)	12.31 (3, 640)	< 0.001	0.99 (0.10)	0.32 (0.19)
Attitudes towards therapist-guided psychological intervention	4.00 (0.71)	4.28 (0.94)	4.37 (0.86)	4.08 (0.84)	4.18 (0.85)	4.39 (3, 641)	< 0.001	− 0.09 (0.10)	− 0.37 (0.19)
Attitudes towards digital self-guided psychological intervention	3.94 (0.63)	4.00 (0.93)	3.83 (0.93)	3.71 (0.84)	3.87 (0.84)	3.47 (3, 641)	0.02	− 0.29 (0.10)	− 0.18 (0.19)
Intention to seek therapist-guided psychological intervention	3.98 (1.04)	4.30 (1.23)	4.50 (1.14)	4.31 (1.19)	4.27 (1.16)	5.34 (3, 641)	= 0.001	− 0.54 (0.10)	− 0.11 (0.19)
Intention to seek digital self-guided psychological intervention	3.63 (1.06)	3.42 (1.37)	3.27 (1.33)	3.31 (1.30)	3.41 (3.33)	4.17 (3, 640)	= 0.053	− 0.05 (0.10)	− 0.80 (0.19)
Depressive symptoms	7.74 (4.70)	11.80 (6.29)	9.57 (5.81)	8.68 (6.06)	9.51 (5.95)	15.03 (3, 641)	< 0.001	0.50 (0.10)	− 0.52 (0.19)

**Table 4:** Zero-order correlations among variables of interest

Variables	Family face concern	Interdependent stigma	Attitudes (therapist-guided)	Attitudes (digital self-guided)	Intention (therapist-guided)	Intention (digital self-guided)	Depressive symptoms
Family face concern	−	0.21***	− 0.07	− 0.02	0.03	0.09*	0.04
Interdependent stigma		−	− 0.24***	− 0.06	− 0.08	0.02	0.25
Attitudes (therapist-guided)			−	0.09*	0.66***	− 0.05	− 0.05
Attitudes (digital self-guided)				−	− 0.02	0.61***	− 0.05
Intention (therapist-guided)					−	0.09*	0.01
Intention (digital self-guided)						−	0.01

***p <.001, *p <.05

**Fig. 2:**
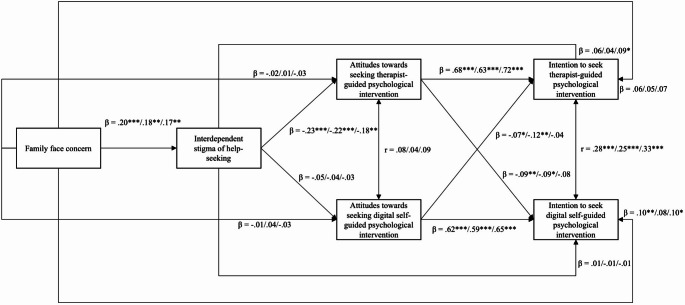
Results of the proposed mediation model. Depressive symptoms were controlled in the model. *p<.05; **p<.01; ***p<.001. Standardized regression coefficient (β) are shown for the overall sample, collectivistic group, and individualistic group, respectively

The results of path analysis (Fig. 2) using the entire sample showed that family face concern was associated with higher levels of interdependent stigma of help-seeking (*β* = 0.20, *p* <.001, 95% CI [0.13, 0.28]). Interdependent stigma of help-seeking was associated with poor attitudes towards seeking therapist-guided psychological intervention (*β* = − 0.23, *p* <.001, 95% CI [−0.31, − 0.16]) but was not significantly associated with attitudes towards seeking digital self-guided psychological intervention (*β* = − 0.05, *p* =.23, 95% CI [−0.13, 0.03]). Attitudes towards seeking therapist-guided psychological intervention was strongly associated with better intention to seek therapist-guided psychological intervention (*β* = 0.68, *p* <.001, 95% CI [0.64, 0.73]) but weakly and significantly associated with intention to seek digital self-guided psychological intervention (*β* = − 0.09, *p* =.004, 95% CI [−0.15, − 0.03]). Similarly, attitudes towards seeking digital self-guided intervention were strongly associated with better intention to seek digital self-guided psychological intervention (*β* = 0.62, *p* <.001, 95% CI [0.57, 0.67]) but weakly and significantly associated with intention to seek therapist-guided psychological intervention (*β* = − 0.07, *p* =.012, 95% CI [−0.13, − 0.02]). Using 5,000 bootstrapped sample, the indirect effect of family face concern on intention to seek therapist-guided psychological intervention through interdependent stigma of help-seeking and attitudes towards therapist-guided psychological intervention was significant (*β* = − 0.03, 95% bootstrapped CI [−0.05, − 0.02]). The indirect effect of family face concern on intention to seek digital self-guided psychological intervention through interdependent stigma of help-seeking and attitudes towards digital self-guided psychological intervention was non-significant (*β* = − 0.01, 95% bootstrapped CI [−0.02, 0.004]). The total indirect effects on intention to seek therapist-guided psychological intervention (*β* = − 0.03, 95% bootstrapped CI [−0.09, 0.02]) and digital self-guided psychological intervention (*β* = − 0.003, 95% bootstrapped CI [−0.05, 0.05]) were non-significant. The direct effect on intention to seek therapist-guided psychological intervention was non-significant (*β* = 0.06, 95% bootstrapped CI [−0.01, 0.12]), whereas that on digital self-guided psychological intervention was significant (*β* = 0.10, 95% bootstrapped CI [0.03, 0.16]).

To further explore potential differences by cultural orientation, path analyses were conducted separately for the collectivistic group (India and Hong Kong; *n* = 315) and the individualistic group (UK and Canada; *n* = 330). The pattern of associations within each group was highly similar to those observed in the overall sample. In both groups, family face concern was significantly associated with higher interdependent stigma of help-seeking (collectivistic group: β = 0.18, *p* =.001, 95% CI [0.07, 0.28]; individualistic group: β = 0.17, *p* =.001, 95% CI [0.07, 0.27]). Interdependent stigma of help-seeking was significantly associated with poorer attitudes towards therapist-guided psychological intervention (collectivistic group: β = − 0.22, *p* <.001, 95% CI [−0.33, − 0.11]; individualistic group: β = − 0.18, *p* =.002, 95% CI [−0.30, − 0.07]), but not significantly associated with attitudes towards digital self-guided intervention in either group (collectivistic group: β = − 0.04, *p* =.56, 95% CI [−0.15, 0.08]; individualistic group: β = − 0.03, *p* =.56,, 95% CI [−0.15, 0.08]). Attitudes towards therapist-guided intervention were strongly associated with intention to seek therapist-guided intervention (collectivistic group: β = 0.63, *p* <.001, 95% CI [0.56, 0.70]; individualistic group: β = 0.72, *p* <.001, 95% CI [0.65, 0.77]), but showed weak to no association with intention to seek digital self-guided intervention (collectivistic group: β = − 0.09, *p* =.045, 95% CI [−0.18, − 0.002]; individualistic group: β = − 0.08, *p* =.08, 95% CI [−0.16, 0.01]). Attitudes towards digital self-guided intervention were strongly associated with intention to seek digital self-guided intervention (collectivistic group: β = 0.59, *p* <.001, 95% CI [0.52, 0.67]; individualistic group: β = 0.65, *p* <.001, 95% CI [0.59, 0.72]) but showed weak to no association with intention to seek therapist-guided intervention (collectivistic group: β = − 0.12, *p* =.005, 95% CI [−0.21, − 0.04]; individualistic group: β = − 0.04, *p* =.35, 95% CI [−0.12, 0.04]).

The indirect effect of family face concern on intention to seek therapist-guided psychological intervention, via interdependent stigma and attitudes towards therapist-guided intervention, was significant in both the collectivistic (β = − 0.02, 95% bootstrapped CI [−0.05, − 0.01]) and individualistic (β = − 0.02, 95% CI [−0.05, − 0.003]) groups. Wald test was not performed as the change in beta was zero, suggesting that the indirect effect operates similarly across collectivistic and individualistic contexts.

The indirect effect of family face concern on intention to seek digital self-guided psychological intervention, via interdependent stigma and attitudes towards digital self-guided psychological intervention, was non-significant in both groups (collectivistic group: β = − 0.004, 95% CI [−0.02, 0.01]; individualistic group: β = − 0.004, 95% CI [−0.02, 0.01]). The total indirect effect on intention to seek therapist-guided psychological intervention was non-significant in both groups (collectivistic group: β = − 0.01, 95% bootstrapped CI [−0.09, 0.07]); individualistic group: β = − 0.03, 95% CI [−0.11, 0.06]). The total indirect effect on the intention to seek digital self-guided psychological intervention was non-significant (collectivistic group: β = 0.02, 95% bootstrapped CI [−0.05, 0.10]); individualistic group: β = − 0.02, 95% CI [−0.09, 0.05]). The direct effect of family face concern on intention to seek digital self-guided intervention was non-significant in the collectivistic group (β = 0.08, 95% CI [−0.01, 0.17]), but was significant in the individualistic group (β = 0.10, 95% CI [0.01, 0.18]). The direct effect of family face concern on intention to seek therapist-guided intervention was non-significant in both groups (collectivistic group: β = 0.05, 95% CI [−0.04, 0.15]; individualistic group: β = 0.07, 95% CI [−0.03, 0.16]).

## Discussion

The present study aimed to investigate the potential mediating roles of interdependent stigma of help-seeking and attitudes towards therapist-guided and digital self-guided psychological interventions in the association between family face concern and the intention to seek corresponding therapist-guided or digital self-guided psychological interventions for depression. The results indicated that family face concern was negatively associated with the intention to seek therapist-guided psychological intervention through increased perception of help-seeking stigma on family members and negative attitudes towards such interventions. Such a mediating effect was not significant for the intention to seek digital self-guided intervention, supporting our speculation that digital self-guided psychological intervention could be less subject to the influence of help-seeking stigma than therapist-guided psychological interventions.

While this finding aligns with previous research that identified a negative association between the concern of losing one’s face and poor help-seeking attitudes and intention [27, 28], it is important to note that family face concern did not significantly correlate with attitudes and intention to seek therapist-guided interventions, as shown by the zero-order correlations. This suggests that although people who are concerned with their family’s face may not directly alter their help-seeking behaviors in therapist-guided interventions, such concern was associated with higher sensitivity to the social stigma that their family members might face if intervention is sought. This increased sensitivity to interdependent help-seeking stigma could potentially influence their attitudes and, consequently, their intention to seek help from therapists.

There are several implications based on the study findings. First, the present study showed that concern of social face of family members could potentially affect one’s intention to seek help. Resonating with the past study [43], the present study highlighted the importance of accounting for one’s perception of how their behaviors might affect their significant others, particularly family members. The findings are consistent with extensive research on self-construal that individuals may define themselves based on their ingroups or people with whom they share close relationships [30, 62]. People with higher interdependent self-construal often take into account how their choices, including the decision to seek help for depression, might affect their family members. Composing of responses from people from four cultural contexts, our study provided evidence that the effect of family face concern on the intention to seek therapist-guided intervention for depression could be generalizable to cultural contexts with varying levels of collectivism.

However, it is important to recognize that cultural generalizability does not imply uniformity. Individuals within any cultural group vary in how strongly they endorse interdependent values or prioritize concerns such as family reputation. While our model appears applicable across cultural contexts with varying degrees of collectivism, interventions should remain sensitive to individual-level cultural values. Efforts to improve help-seeking attitudes and intentions may benefit from culturally tailored messaging and psychoeducation that explicitly address family- and relationship-centered concerns. Prior research has demonstrated the importance of family involvement in this regard. For instance, Yu et al. (2021) found that involving family members can enhance recovery-oriented services by strengthening personal narratives [63]. Similarly, it was showed that family support can help mitigate the negative impact of mental health stigma [64]. Encouraging open communication and supportive family engagement may, in turn, help ease stigma and alleviate concerns related to family face.

Culturally adapted interventions, including but not limited to adapting educational materials to reflect the language, values, and social expectations of the target population, increases coping skills and reduced perceived stigma, and enhanced trust in mental health services among the users [65, 66]. Delivering messages that normalize concerns about family reputation or address common misconceptions may also help reduce interdependent stigma, and thereby foster more positive attitudes, and ultimately increase individuals’ intentions to seek help for mental health concerns.

Nevertheless, while the observed indirect effect was statistically significant but small (β = − 0.03), it is essential to interpret this finding within the broader context of stigma and help-seeking intentions. Focusing solely on individual-level processes, such as interdependent stigma and family face concern, captures only part of the broader context that determines help-seeking behavior. For instance, many barriers to seeking help are systemic, including limited availability of services or financial constraints [67, 68]. These barriers can impede help-seeking intention regardless of an individual’s family face concern, interdependent stigma, or attitude towards treatment. In addition, public stigma against people seeking help can operate independently of personal beliefs. Individuals who are ready to seek help may still have the barriers in doing so due to anticipated social consequences, including labelling, exclusion, or anticipated stigma [15]. Therefore, the small effect size observed in the present study may reflect the constrained role of individual-level stigma when larger structural and social forces are at play. This is consistent with suggested strategies that target not only self-stigma but public and structural stigma through different strategies such as policy change and facilitation of mental health literacy [67]. Effective interventions should address not only personal attitudes and beliefs but also public discourse, institutional policies, and systemic inequities. Public education campaigns, workplace mental health initiatives, anti-discrimination laws, and improvements in service accessibility and cultural responsiveness are all critical components of broader stigma reduction efforts.

Lastly, findings of the present study indicate that intention to seek digital self-guided psychological intervention may be less affected by concern regarding family face than is the case with therapist-guided psychological intervention. This finding is understandable, considering that digital self-guided interventions can be accessed privately and at one’s own discretion. Consequently, individuals engaging in digital interventions are at a lower risk of being found out by others, which in turn reduces the impact of help-seeking stigma that often discourages help-seeking [39]. Thus, even if individuals are concerned about the potential stigma their family might face should they seek help, this concern is less pertinent to digital self-guided psychological interventions. This insight suggests that digital interventions might offer a culturally sensitive alternative for those who might be reluctant to seek in-vivo therapist-guided interventions due to worries about family reputation and help-seeking stigma.

The accessibility and privacy of digital self-guided interventions may provide a discreet avenue for help-seeking, which could be particularly appealing to those who are sensitive to the opinions and judgments of their social circle. This could have significant implications for mental health service delivery, especially in cultures or communities where stigma is a major barrier to seeking help [26]. Additionally, digital solutions such as online psychoeducation, telehealth, and self-guided interventions have been found to be effective in reducing stigma and supporting mental health, and should be considered [69–72]. However, consistent with the previous review that people in general have greater preference and intention to engage face-to-face over digital mental health interventions [73], the present study observed that the mean levels of attitudes and intention to seek therapist-guided psychological intervention indeed are larger than digital self-guided intervention. While the results imply that therapist-guided interventions remain to be a preferred means of support for people with depressive symptoms, given the increasing scientific evidence demonstrating the efficacy and effectiveness of varied digital psychological interventions, the findings highlighted the need to psychoeducate individuals about the evidence and benefits of digital self-guided psychological interventions so that they can become a viable option for people to choose from.

With millennials and Gen Z being digital natives and digital technology becoming a major part of living in the majority of the global population, the delivery of evidence-based mental health services could leverage this prevailing technology to popularize and increase access to this viable option widely [74], especially for those who might otherwise forgo treatment due to fear of social repercussions. However, it is important to note that the present study only investigated the effect of family face concern and interdependent stigma of help-seeking on attitudes and intention to seek digital self-guided interventions. As previous studies have shown that personal stigma about depression could be negatively associated with intention to seek Internet-based psychological intervention [75], future work comparing the effects between personal and family face concern and self- and interdependent help-seeking stigma on intention to seek digital self-guided psychological interventions is warranted.

This study has several points that warrant attention when interpreting the results. First, the data collected is cross-sectional in nature. Therefore, no causality should be inferred. Second, although we collected the data from four cultural contexts, they were primarily from college students and women. This could limit the generalizability of the findings. Furthermore, the present study did not separately analyze the mediating models in four cultural contexts but instead examined the variability of family face concern and interdependent stigma of help-seeking by group the individualistic and collectivistic group due to power and sample size considerations. Future studies may consider separately testing the mediating model across cultural contexts and across ethnic groups, especially regions such as Canada and UK where participants reported diverse ethnic backgrounds. However, given the sample size for each ethnic group is small, separate analyses were not possible for the present study. Third, we did not further divide the therapist-guided psychological interventions and digital self-guided psychological interventions into subcategories. Future studies may further examine how family face concern and interdependent stigma might have differential effects on attitudes and intention of different types and forms of interventions [76]. Lastly, it is also important to note that a few indicators of goodness-of-fit (e.g., RMSEA) for the Family Face Concern Scale showed only marginal fit. This may indicate the need for further validation of the scale, possibly with a larger and more cross-cultural sample. However, we contend that the new conceptualization of family face concern provides valuable evidence of how a novel cultural characteristic can be related to stigma, thereby contributing to the development of the literature on culture and stigma.

Despite these potential limitations, the present study provides evidence supporting the potential effects of family face concern on individuals’ intention to seek therapist-guided and digital self-guided psychological interventions. While concerns about family face and relational stigma may serve as the indirect barriers to seeking traditional therapist-guided intervention, digital self-guided interventions appear less constrained by such concerns. This result highlighted the potential of digital intervention as an accessible and less stigma-sensitive option, particularly for individuals or communities in which family honor and social reputation strongly shape help-seeking behavior. Taken together, these results elucidate the roles of family face concern, interdependent stigma, and modality in mental health engagement across cultures with varying levels of collectivism, which is an essential step toward more inclusive and effective care.

## Data Availability

The data are available from the authors upon reasonable request.
